# Pilot evaluation of a novel, automated ergonomics assessment tool

**DOI:** 10.1055/a-2568-9610

**Published:** 2025-05-12

**Authors:** Bara El Kurdi, Sumbal Babar, Ali Soroush, Jay Bapaye, Reid D. Wasserman, Juan Echavarria, Omer Shahab, Cameron Locke, Jamie Yang, Michael Koachman, Klaus Mönkemüller, Aasma Shaukat

**Affiliations:** 1 6912 Gastroenterology, Carilion Clinic, Roanoke, United States; 2 6912 Infectious Diseases, Carilion Clinic, Roanoke, United States; 3 5925 Gastroenterology, Mount Sinai School of Medicine, New York, United States; 4 6912 Internal Medicine, Carilion Clinic, Roanoke, United States; 5 14742 Division of Gastroenterology, Hepatology, and Nutrition, The University of Texas Health Science Center at San Antonio, San Antonio, United States; 6 1305 Gastroenterology, Virginia Hospital Center Arlington Health System, Arlington, United States; 7 12297 Gastroenterology, NYU Langone Health, New York, United States; 8 8783 Gastroenterology, UCLA, Los Angeles, United States; 9 14640 Gastroenterology, University of Pennsylvania Perelman School of Medicine, Philadelphia, United States; 10 243516 Gastroenterology, HELIOS Frankenwald Hospital Kronach, Kronach, Germany

**Keywords:** Endoscopy Upper GI Tract, Endoscopy Lower GI Tract, Endoscopy Small Bowel

## Abstract

**Background and study aims:**

Gastroenterologists are prone to endoscopy-related musculoskeletal injuries (ERI). Current interventions lack real-time monitoring and feedback. ErgoGenius, a novel artificial intelligence computer-vision tool, addresses this gap by providing continuous posture assessment and feedback without wearable motion trackers. The aim of this study was to determine the feasibility of ErgoGenius, its accuracy compared with human appraisers, and its ability to detect abnormal posture.

**Methods:**

The study was conducted at two large academic centers. The Rapid Entire Body Assessment (REBA) score was used as a surrogate for ergonomic performance and risk of injury. Ten endoscopists of varying gender, height, and weight were recorded performing endoscopic tasks in optimal vs. lowered bed positions. Videos were analyzed by ErgoGenius. A paired *t* -test was used to compare REBA scores between bed positions.

**Results:**

ErgoGenius was successfully deployed in a controlled endoscopy setting. ErgoGenius achieved perfect internal agreement (rho = 1) and closely correlated with human appraisers (rho = 0.987). Average REBA scores were notably higher in the lowered bed position (mean 4.64) compared with the optimal position (mean 2.55), ( *P* = 0.006).

**Conclusions:**

ErgoGenius was successfully deployed to detect abnormal postures related to changes in bed position and quantify ERI risk. It performed at par with human appraisers. This tool shows promise in enhancing ergonomic practices among gastroenterologists and trainees, potentially leading to better health outcomes and reduced injury.

## Introduction

Gastroenterologists spend long hours in the endoscopy suite performing ever-higher volumes of complex procedures with little rest or recovery. In 2006, it was reported that on average, endoscopists performed 12 upper endoscopies and 22 colonoscopies per week 1 . More recent reports have shown that some endoscopists perform as many as 24 endoscopic procedures per day 2 . Such a demanding lifestyle predisposes endoscopists to injury. In a recent survey of 1,698 gastroenterologists, 75% reported experiencing endoscopy-related musculoskeletal injuries (ERIs) 3 . This topic has garnered a significant amount of attention, including ergonomics-related publications 4 5 6 7 8 , instructional videos 9 10 11 , and yearly talks at national meetings and fellowship programs. Summarizing this work, the American Society of Gastrointestinal Endoscopy recently published endoscopy ergonomics guidelines 12 . Although these efforts are essential, education and awareness alone are inadequate for meaningful improvement in ergonomics for a variety of reasons 13 ; citing forgetfulness, busy endoscopy schedule, and lack of insight, endoscopists often revert to old patterns of behavior despite viewing and understanding the ergonomics educational materials. Passive lecture-based didactics are poorly suited to break habits formed over years of training and practice because they do not provide real-time coaching or recurring ergonomic performance feedback.

To fill this gap, we developed ErgoGenius, a computer-vision tool that provides live posture assessment, comprehensive posture analysis, and longitudinal feedback for healthcare providers and trainees. The tool analyzes video capture data of endoscopist movements, without the need for wearable sensors. Utilizing artificial intelligence (AI)-based posture analysis, it identifies relevant joint locations to establish posture and calculate the validated Rapid Entire Body Assessment (REBA) ergonomic score 14 15 16 17 ; a validated surrogate for musculoskeletal injury risk. ErgoGenius provides a personalized procedure-level ergonomics analysis of each major joint that is coupled with tailored feedback on improving posture. ErgoGenius is deployed in real time, providing live feedback, and offering longitudinal monitoring to follow the results of each intervention over time. The aims of this study were to determine the feasibility of deploying ErgoGenius in a controlled environment in the endoscopy suite, its accuracy compared with independent human appraisers, and its ability to detect abnormal posture.

## Methods

### Software

ErgoGenius employs a convolutional neural network to detect body parts from still images and is based after a combination of the BlazePose 18 , BlazeFace 19 , and the single-shot-detector 20 models. The software is designed to determine postural changes in real-time. ErgoGenius can process videos with up to 60 frames per second. It analyses posture in each frame separately and averages out the scores over all frames. The software has been tailored to the types of movements often performed in the endoscopy suite such as neck flexion, extension, rotation, trunk movements including bending and rotation, upper extremity movements including torque, as well as lower extremity position and weight loading.

### Study type

This was a prospective, open label, dual center, pilot study.

### Set-up

The study took place in the endoscopy suites of two academic medical centers (University of Texas in San Antonio and New York University Langone Health). The participants were gastroenterology fellows in years 1 to 3 of training. The participants performed a limited endoscopic challenge using the Olympus Thompson Box (the task consisted of using rat-tooth forceps to pick up and transfer plastic shapes from one end of the box to the other requiring retroflexion to place the plastic shapes on top of plastic spikes protruding on either side of the box). Participants were filmed using an iPhone12 rear-view camera. Each participant performed the same 2-minute challenge in two different settings. The first was performed with the bed in optimal position (5–10 cm below elbow height). The second was performed with the bed lowered to about 1 to 5 inches above knee height (this depended on endoscopist height and limitations of bed descension).

Videos were collected and fed into the ErgoGenius tool. Procedure-level mean Rapid Entire Body Assessment (REBA) scores were calculated. REBA is calculated by examining various factors such as joint positioning and force exertion 14 21 . Scores are separately assigned for neck, trunk, arm, wrist, and lower limb postures (Supplementary Fig. 1).

### Outcomes

#### Validation

To determine the accuracy of ErgoGenius REBA calculations, we compared them to the current gold standard (human appraisal). Four independent providers from three institutions participated in this process. In the beginning, the participants were introduced to the REBA scoring system, and were provided with examples of how to calculate it. They were then given 10 randomly chosen screenshots of endoscopists performing endoscopic procedures. They independently calculated REBA scores for the endoscopist in each of these images (Supplementary Fig. 1). The images were later processed by ErgoGenius with three repetitions. The similarity between ErgoGenius and human REBA scores was compared using Spearman’s rank correlation coefficient.

Ergonomic performance based on bed position:

Our primary endoscopist outcome was the change in REBA scores with change in bed position. A paired T-test was used to compare REBA scores for each bed position. *P* < 0.05 was considered statistically significant.

## Results

Ten providers (five male and five female) of different heights and weights participated in our study ( Table 1 ). ErgoGenius was successfully deployed in a controlled endoscopy setting.

**Table:** **Table 1** Endoscopist characteristics.

Endoscopist	Sex	Height	Weight
**1**	M	185	198
**2**	F	165	140
**3**	M	180	186
**4**	M	193	235
**5**	F	160	140
**6**	F	165	140
**7**	F	168	145
**8**	F	160	128
**9**	M	185	175
**10**	M	167	145

### Validation

**Fig. 1:**
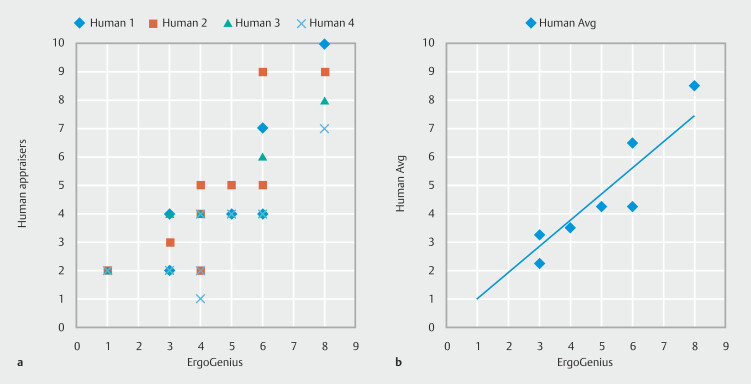
**a** Correlation of human-appraiser scores with ErgoGenius-generated scores. **b** Correlation of average human-appraiser scores with ErgoGenius scores.

Four independent appraisers participated in the validation cohort. Human appraisers had relatively poor internal agreement on REBA scores with an average rho of 0.71, whereas ErgoGenius had perfect internal agreement with rho of 1. The average readings of human appraisers correlated to those of ErgoGenius with a rho of 0.987, indicating that ErgoGenius performs at the level of human appraisers and validating its accuracy for this task ( Fig. 1 , Supplementary Table 1).

### Ergonomic performance based on bed position

**Fig. 2:**
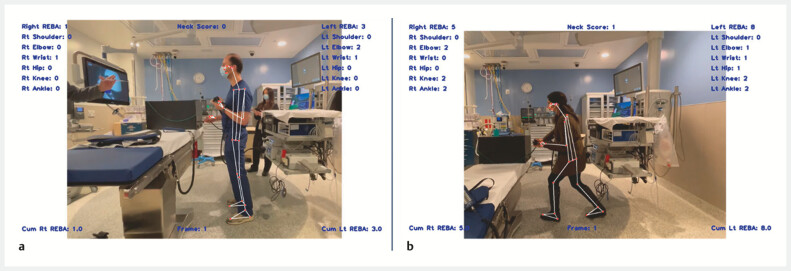
ErgoGenius screen showing real-time comprehensive ergonomic analysis. **a** Endoscopist in semi-neutral posture with low-risk scores. **b** Endoscopist in contorted posture with crouching and twisting movements, resulting in higher-risk scores.

A significant decline in ergonomic performance (indicated by increased REBA scores) occurred when the bed was switched to the lower position with a mean difference of 2.1 ( *P* = 0.006) ( Table 2 , Fig. 2 ).

**Table:** **Table 2** REBA scores based on bed height.*

Endoscopist	Bed height	
	Optimal	Low
**1**	2.58	2.62
**2**	2.67	4.6
**3**	3.54	5.27
**4**	1.68	4.47
**5**	2.26	9.14
**6**	2.62	3.87
**7**	3.15	5.14
**8**	2.29	3.29
**9**	1.97	3.55
**10**	2.72	4.48
**Mean**	2.548	4.643
***P* value **	**0.0055**	
*Ergonomic scores for participants at optimal bed height (bed height 5–10 cm below elbow height) and low bed height (bed height up to slightly above knee position). Higher scores correspond to higher risk of injury.		

## Discussion

We validated a novel automated ergonomics assessment in a controlled setting in the endoscopy suite. It was able to detect abnormal posture related to change in bed position and exhibited human-level accuracy in calculating REBA scores that reflect risk of musculoskeletal injury, showing promise as a novel method of automating ergonomic assessment for endoscopists in the real world in an objective way.

Up to 75% of gastroenterologists suffer from ERI 3 , which undermines their welfare and productivity; 28% of endoscopists reduce their procedure volume due to pain, 25% increase time between procedures to avoid pain, 65% use nonsteroidal anti-inflammatory drugs to treat musculoskeletal pain, 37% reduce physical activity outside of work to avoid pain, and 10% report missing days from work due to pain (median 30 days). These numbers only reflect part of the problem because musculoskeletal injury results in reduced access to healthcare for patients whose providers are sidelined due to injury. A cost analysis conducted by our group showed that a missed day for a gastroenterologist translates into $15,760 in lost revenue, and a drop in case volume of 25% translates into $78,700 in lost revenue per month for a gastroenterology practice. These numbers reflect the economic impact of the problem on a gastroenterology practice. While no data currently exist on rates of forced retirement as a result of musculoskeletal injury for endoscopists, a recent study investigating the effects of musculoskeletal injury on interventional medical specialties reported that 12% of physicians with work-related musculoskeletal injuries required a leave of absence, practice restriction, or early retirement 22 . Existing measures to address this problem lack real-time continuous monitoring, a key feature offered by ErgoGenius. Previous studies have shown that training programs and personal assessment by ergonomics experts lead to lower risk of injury 6 23 . However, access to these experts is extremely limited. ErgoGenius offers an automated and accessible alternative, dramatically increasing provider access to ergonomic assessment and expertise both in urban and rural areas.

Our study was limited by the relatively small sample size and use of a Thompson box challenge rather than a clinical patient-based procedure. However, as a pilot study, we provide robust data showing utility and clear statistical significance despite the small sample size. Although real patients were not included, the study was conducted in two clinical endoscopy suites often used for clinical procedures rather than a sim-lab, further supporting usability in real-life clinical setting. Future studies including larger sample sizes conducted in patient-care settings are needed to revalidate our results.

Our findings suggest that AI- and computer-vision based technologies may have a role to play in the clinical environment in a provider-focused manner. We find that ErgoGenius may be able to function as an ergonomics trainer, providing real-time monitoring and risk assessment. Future iterations of this technology can focus on more detailed analysis of the endoscopy suite environment, set-up, and equipment and identify further risks. In addition, this technology may be used to appraise the potential ergonomic impact of new endoscopic technologies and guide manufacturers to adopt more ergonomically friendly designs earlier in the device development process.

## Conclusions

ErgoGenius was able to detect abnormal posture and quantify ERI risk by calculating the validated REBA score for providers in real time at the level of human appraisers. Future studies are needed to study the effectiveness of this tool in reducing risk of injury among providers by identifying their areas of improvement from an ergonomic standpoint and offering real-time and longitudinal feedback.

## Notes

Correction **Pilot evaluation of a novel, automated ergonomics assessment tool** Bara El Kurdi, Sumbal Babar, Ali Soroush et al. Endoscopy International Open 2025; 13: a25689610. DOI: 10.1055/a-2568-9610 In the above-mentioned article the legends of Figure 1 and Figure 2 have been corrected. This was corrected in the online version on 04.06.2025.

## Supplementary Material

Supplementary Material Zusatzmaterial
