# Correction: Pilot evaluation of a novel, automated ergonomics assessment tool

**DOI:** 10.1055/a-2625-8430

**Published:** 2025-06-04

**Authors:** Bara El Kurdi, Sumbal Babar, Ali Soroush, Jay Bapaye, Reid D. Wasserman, Juan Echavarria, Omer Shahab, Cameron Locke, Jamie Yang, Michael Koachman, Klaus Mönkemüller, Aasma Shaukat

**Affiliations:** 1 6912 Gastroenterology, Carilion Clinic, Roanoke, United States; 2 6912 Infectious Diseases, Carilion Clinic, Roanoke, United States; 3 5925 Gastroenterology, Mount Sinai School of Medicine, New York, United States; 4 6912 Internal Medicine, Carilion Clinic, Roanoke, United States; 5 14742 Division of Gastroenterology, Hepatology, and Nutrition, The University of Texas Health Science Center at San Antonio, San Antonio, United States; 6 1305 Gastroenterology, Virginia Hospital Center Arlington Health System, Arlington, United States; 7 12297 Gastroenterology, NYU Langone Health, New York, United States; 8 8783 Gastroenterology, UCLA, Los Angeles, United States; 9 14640 Gastroenterology, University of Pennsylvania Perelman School of Medicine, Philadelphia, United States; 10 243516 Gastroenterology, HELIOS Frankenwald Hospital Kronach, Kronach, Germany



In the above-mentioned article the legends of Figure 1 and Figure 2 have been corrected. This was corrected in the online version on 04.06.2025.

