# Supplementary material for: Pilot evaluation of a novel, automated ergonomics assessment tool
Source: Endosc Int Open. 2025 May 12;13:a25689610. doi: 10.1055/a-2568-9610 (PMC12080516; doi:10.1055/a-2568-9610)
Supplement: Supplementary file 1 — Supplementary Material [file 10-1055-a-2568-9610_25710350.pdf]

Supplementary Table 1 Spearman rank coefficient of human scores to AI scores.

	Human 1	Human 2	Human 3	Human 4	Human Avg	ErgoGenius
Human 1	1.000	0.785	1.000	0.632	0.898	0.848
Human 2	0.785	1.000	0.785	0.453	0.874	0.861
Human 3	1.000	0.785	1.000	0.632	0.898	0.848
Human 4	0.632	0.453	0.632	1.000	0.768	0.771
Human Avg	0.898	0.874	0.898	0.768	1.000	0.987
ErgoGenius	0.848	0.861	0.848	0.771	0.987	1.000

AI, artificial intelligence.

Supplementary Fig. 1 REBA score calculation sheet.

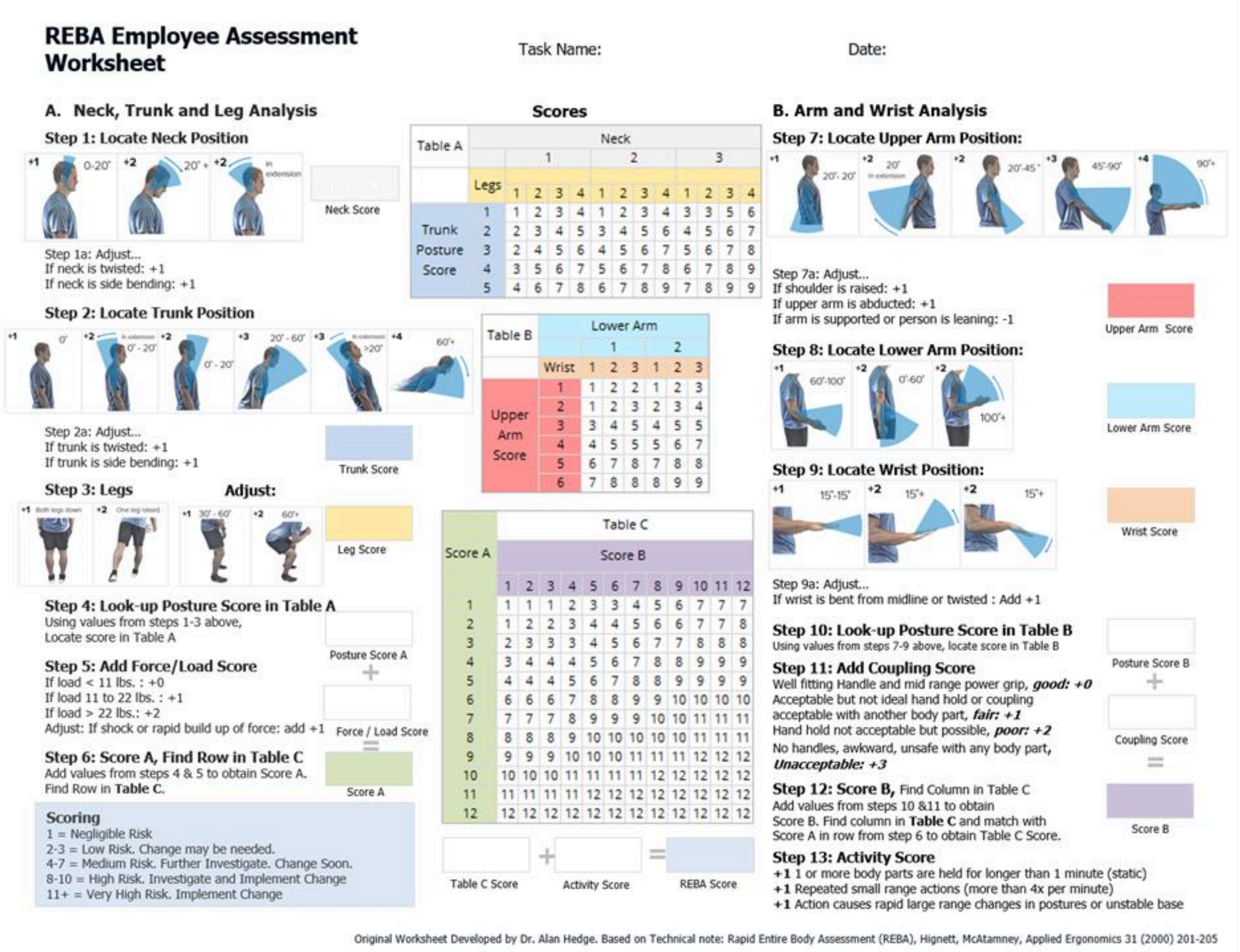

REBA Employee Assessment Worksheet

Task Name:

Date:

A. Neck, Trunk and Leg Analysis

Step 1: Locate Neck Position

+1 0-20° | +2 20°+ | +2 in extension

Neck Score

Step 1a: Adjust…
If neck is twisted: +1
If neck is side bending: +1

Step 2: Locate Trunk Position

+1 0° | +2 in extension 0°-20° | +2 0°-20° | +3 20°-60° | +3 in extension >20° | +4 60°+

Step 2a: Adjust…
If trunk is twisted: +1
If trunk is side bending: +1

Trunk Score

Step 3: Legs **Adjust:**

+1 Both legs down | +2 One leg raised | +1 30°-60° | +2 60°+

Leg Score

Step 4: Look-up Posture Score in Table A
Using values from steps 1-3 above,
Locate score in Table A

Posture Score A
+

Step 5: Add Force/Load Score
If load < 11 lbs. : +0
If load 11 to 22 lbs. : +1
If load > 22 lbs.: +2
Adjust: If shock or rapid build up of force: add +1

Force / Load Score
=

Step 6: Score A, Find Row in Table C
Add values from steps 4 & 5 to obtain Score A.
Find Row in **Table C.**

Score A

Scoring
1 = Negligible Risk
2-3 = Low Risk. Change may be needed.
4-7 = Medium Risk. Further Investigate. Change Soon.
8-10 = High Risk. Investigate and Implement Change
11+ = Very High Risk. Implement Change

Scores

Table A	Neck	1				2				3			
	Legs	1	2	3	4	1	2	3	4	1	2	3	4
Trunk Posture Score	1	1	2	3	4	1	2	3	4	3	3	5	6
	2	2	3	4	5	3	4	5	6	4	5	6	7
	3	2	4	5	6	4	5	6	7	5	6	7	8
	4	3	5	6	7	5	6	7	8	6	7	8	9
	5	4	6	7	8	6	7	8	9	7	8	9	9

Table B	Lower Arm	1			2		
	Wrist	1	2	3	1	2	3
Upper Arm Score	1	1	2	2	1	2	3
	2	1	2	3	2	3	4
	3	3	4	5	4	5	5
	4	4	5	5	5	6	7
	5	6	7	8	7	8	8
	6	7	8	8	8	9	9

Table C<br>Score A	Score B 1	2	3	4	5	6	7	8	9	10	11	12
1	1	1	1	2	3	3	4	5	6	7	7	7
2	1	2	2	3	4	4	5	6	6	7	7	8
3	2	3	3	3	4	5	6	7	7	8	8	8
4	3	4	4	4	5	6	7	8	8	9	9	9
5	4	4	4	5	6	7	8	8	9	9	9	9
6	6	6	6	7	8	8	9	9	10	10	10	10
7	7	7	7	8	9	9	9	10	10	11	11	11
8	8	8	8	9	10	10	10	10	10	11	11	11
9	9	9	9	10	10	10	11	11	11	12	12	12
10	10	10	10	11	11	11	11	12	12	12	12	12
11	11	11	11	11	12	12	12	12	12	12	12	12
12	12	12	12	12	12	12	12	12	12	12	12	12

Table C Score + Activity Score = REBA Score

B. Arm and Wrist Analysis

Step 7: Locate Upper Arm Position:

+1 20°-20° | +2 20° in extension | +2 20°-45° | +3 45°-90° | +4 90°+

Step 7a: Adjust…
If shoulder is raised: +1
If upper arm is abducted: +1
If arm is supported or person is leaning: -1

Upper Arm Score

Step 8: Locate Lower Arm Position:

+1 60°-100° | +2 0°-60° | +2 100°+

Lower Arm Score

Step 9: Locate Wrist Position:

+1 15°-15° | +2 15°+ | +2 15°+

Wrist Score

Step 9a: Adjust…
If wrist is bent from midline or twisted : Add +1

Step 10: Look-up Posture Score in Table B
Using values from steps 7-9 above, locate score in Table B

Posture Score B
+

Step 11: Add Coupling Score
Well fitting Handle and mid range power grip, ***good: +0***
Acceptable but not ideal hand hold or coupling acceptable with another body part, ***fair: +1***
Hand hold not acceptable but possible, ***poor: +2***
No handles, awkward, unsafe with any body part, ***Unacceptable: +3***

Coupling Score
=

Step 12: Score B, Find Column in Table C
Add values from steps 10 &11 to obtain Score B. Find column in **Table C** and match with Score A in row from step 6 to obtain Table C Score.

Score B

Step 13: Activity Score
+1 1 or more body parts are held for longer than 1 minute (static)
+1 Repeated small range actions (more than 4x per minute)
+1 Action causes rapid large range changes in postures or unstable base

Original Worksheet Developed by Dr. Alan Hedge. Based on Technical note: Rapid Entire Body Assessment (REBA), Hignett, McAtamney, Applied Ergonomics 31 (2000) 201-205
